# New Insights into the Nature of Cerebellar-Dependent Eyeblink Conditioning Deficits in Schizophrenia: A Hierarchical Linear Modeling Approach

**DOI:** 10.3389/fpsyt.2016.00004

**Published:** 2016-01-25

**Authors:** Amanda R. Bolbecker, Isaac T. Petersen, Jerillyn S. Kent, Josselyn M. Howell, Brian F. O’Donnell, William P. Hetrick

**Affiliations:** ^1^Department of Psychological and Brain Sciences, Indiana University, Bloomington, IN, USA

**Keywords:** schizophrenia, eyeblink conditioning, cerebellum, associative learning, reflex conditioning, conditioned response, cognition, psychosis

## Abstract

Evidence of cerebellar dysfunction in schizophrenia has mounted over the past several decades, emerging from neuroimaging, neuropathological, and behavioral studies. Consistent with these findings, cerebellar-dependent delay eyeblink conditioning (dEBC) deficits have been identified in schizophrenia. While repeated-measures analysis of variance is traditionally used to analyze dEBC data, hierarchical linear modeling (HLM) more reliably describes change over time by accounting for the dependence in repeated-measures data. This analysis approach is well suited to dEBC data analysis because it has less restrictive assumptions and allows unequal variances. The current study examined dEBC measured with electromyography in a single-cue tone paradigm in an age-matched sample of schizophrenia participants and healthy controls (*N* = 56 per group) using HLM. Subjects participated in 90 trials (10 blocks) of dEBC, during which a 400 ms tone co-terminated with a 50 ms air puff delivered to the left eye. Each block also contained 1 tone-alone trial. The resulting block averages of dEBC data were fitted to a three-parameter logistic model in HLM, revealing significant differences between schizophrenia and control groups on asymptote and inflection point, but not slope. These findings suggest that while the learning rate is not significantly different compared to controls, associative learning begins to level off later and a lower ultimate level of associative learning is achieved in schizophrenia. Given the large sample size in the present study, HLM may provide a more nuanced and definitive analysis of differences between schizophrenia and controls on dEBC.

## Introduction

Schizophrenia is a complex disorder with diverse symptoms and heterogeneous expression. Besides its cardinal psychotic symptoms, cognitive and motor abnormalities are prominent symptoms of the disorder. The cognitive dysmetria theory of schizophrenia (1) provides a unitary framework that can account for the disparate symptoms of schizophrenia. It posits that disruptions in the cortico–cerebello–thalamo–cortical circuit (CCTCC) lead to poor coordination of information, resulting in different symptom constellations. Given that the cerebellum plays a role in temporal processing (2), it may occupy a unique role in this circuit by modulating the temporal coordination of information. Consistent with this proposition, evidence collected over the last several decades points to not only an important cerebellar role in coordinated movement and motor learning, but also non-motor psychological processes, most notably cognition (3–8). The neuroanatomical substrate for these functional effects has been revealed by studies confirming that the CB is reciprocally connected to prefrontal, parietal, and motor/premotor cortex (9–12). It is not surprising then that lesions to the cerebellum can produce symptoms commonly seen in schizophrenia, including visuospatial deficits, attention deficits, executive dysfunction, flattened affect, disinhibited, and socially inappropriate behavior (6).

Neuropathological and neuroimaging studies have documented morphological and functional cerebellar abnormalities in schizophrenia. For example, subjects with schizophrenia have reduced bilateral cerebellar volume (13), abnormal cerebellar connectivity to cerebral regions involved in both motor and cognitive functions (14), cerebellar morphological abnormalities (15), and reductions in Purkinje cell size and density (16–18). Even groups at clinical and familial risk for psychosis show reduced cerebellar gray matter (19) compared to non-risk groups. However, negative findings exist both in the neuroimaging (20) and neuropathology (21) literature.

Importantly, first-episode (22–24) and antipsychotic medication naïve schizophrenia patients (25) have reduced cerebellar volume, suggesting that cerebellar abnormalities are characteristic of the disorder rather than medication use. Perhaps most convincingly, cerebellar volume is associated with cognitive deficits (26) as well as symptoms of depression, negative symptoms, and psychotic features in schizophrenia (25, 27, 28), suggesting that illness severity or progression may coincide with cerebellar degradation.

Delay eyeblink conditioning (dEBC) is an associative learning task that is highly dependent upon cerebellar functioning (29–31), in. The neuro-circuitry of this task has been extensively studied, and evidence overwhelmingly supports the conclusion that the cerebellum is critical both for learning the association between the unconditioned and conditioned stimuli and for the expression of the conditioned eyeblink response (32, 33). Numerous additional brain regions (i.e., hippocampus, medial septum, frontal cortex) can change the way in which the eyeblink response is expressed (34), neuroplasticity in the cerebellum initially elicits the classically conditioned eyeblink response (35).

Over the past decade, accumulating evidence indicates that cerebellar-mediated dEBC associative learning is abnormal in schizophrenia (36–38), schizotypal personality disorder (39), and first-degree relatives of schizophrenia patients (38). These associative learning deficits in schizophrenia may be remediated by pharmacological intervention (37).

One outstanding issue in the dEBC literature is statistical in nature. Specifically, a repeated-measures analysis of variance (ANOVA) is commonly used to analyze dEBC data, despite the availability of superior and more sophisticated statistical techniques, such as hierarchical linear modeling (HLM), which may reveal more reliable and nuanced findings. In our previous studies of dEBC using ANOVA, we have found conflicting results with respect to whether the learning rate (e.g., the block by group interaction in ANOVA) differs between groups. Several studies have found that the schizophrenia group had a reduced acquisition rate (36, 39), while others found no difference between groups (38, 40). Notably, the study with the largest sample size (*N* = 62) found a reduced average percentage of conditioned responses from subjects with schizophrenia, but no between-group differences in acquisition rate compared to healthy controls (40).

Hierarchical linear modeling is particularly well suited to dEBC data analysis and is superior to repeated-measures ANOVA for measuring time-dependent change because it takes into consideration the statistical dependencies in repeated-measures designs. HLM can be considered a special case of regression that can accommodate variance on more than one level (i.e., nested data), in this case, at both the individual level and at the group level. In HLM, the best-fitting line for each individual is identified, but each line fit is also influenced by the trajectories of other group members. This aspect of HLM has the effect of increasing the accuracy of each individual’s fit while minimizing the error of measurement at the individual and group level. Moreover, HLM has less restrictive assumptions, can tolerate missing data points, and can accommodate hierarchical or nested data structures (41). Perhaps the greatest strength of HLM is that heterogeneity of variance is treated as potentially meaningful information that can help to identify significant interactions between variables (42), whereas in ANOVA it is treated as a nuisance factor. Finally, HLM can be used to examine growth curves that model traditional learning curves so that important parameters, such as the slope, asymptote, and inflection point of the fitted curves can be quantified. [For a more comprehensive explanation of the use of HLM in repeated-measures designs, please see Ref. (43)].

Hierarchical linear modeling was implemented in a recent study (44, 45) in which dEBC data from healthy controls, individuals with schizophrenia, and first-degree relatives of individuals with schizophrenia (*N* = 18 per group) were fitted to a linear model. Differences in acquisition rate (i.e., slope), indicating a slower rate of associative learning was found between both the schizophrenia and family members groups compared to controls. In the present study, data from a larger schizophrenia sample was age-matched to controls (*N* = 59 per group) and HLM was applied to a three-parameter logistic growth model to more closely approximate a learning curve. We predicted that the slope of the learning curve would be lower for the schizophrenia group, indicating a slower learning rate. We also expected that the asymptote – the maximum level of performance – would be lower in schizophrenia, and that the inflection point, which is the point on the learning curve when learning begins to slow down and level off, would occur later.

## Materials and Methods

### Participants

Participants were 56 individuals (17 females) who were diagnosed with schizophrenia and 56 age-matched control participants (29 females). Control participants had no history of psychotic and mood disorders and no history of schizophrenia spectrum disorders within first-degree relatives. Data from 36 individuals with schizophrenia (12 females) and 32 controls (15 females) included in this study had been included in an earlier study of dEBC that used more traditional analysis methods (40). Participants with schizophrenia were recruited through outpatient and inpatient units at local hospitals. The control group was recruited by posting community and newspaper advertisements. Participants’ demographic, clinical, and medication information can be seen in Table 1. Welch’s *t*-test showed that, as expected due to age-matching, the mean age of schizophrenia participants did not differ from controls [*t*(1,112) = −0.29, *P* = 0.77]. Sex was significantly different across groups [χ2(1) = 4.46, *P* = 0.035], with more males in the schizophrenia group (see Table 1). Importantly, sex was used as a covariate in the HLM analyses and it did not significantly improve model fit (*p* > 0.05).

**Table 1:** **Demographic, clinical, and medication information**.

	Schizophrenia	Controls
Age (years)	*M* = 36.4 (SD = 10)	*M* = 35.8 (SD = 10)
Sex (M:F)	39:17	27:29
PANSS total score	*M* = 59 (SD = 13)	–
*Positive*	*M* = 16 (SD = 6)	–
*Negative*	*M* = 15 (SD = 5)	–
*General*	*M* = 28 (SD = 6)	–
^a^Past alcohol dependence	13	0
Past illicit drug dependence	16	0
^b^Psychotropic medication		
*No antipsychotic medication*	6	56
*Atypical antipsychotic*	44	0
*Typical antipsychotic*	12	0

*^a^Nine schizophrenia patients met criteria for both past alcohol and other drug dependence*.

*^b^Eight schizophrenia patients were taking both typical and atypical antipsychotic drugs at the time of testing. Medication information was not available for two participants with schizophrenia*.

The Diagnostic and Statistical Manual of Mental Disorders-IV Axis I Disorders (SCID-I) (46) sections for mood disorders, psychotic disorders, and substance abuse disorders was used to diagnose participants in the schizophrenia group. Medical records were consulted to refine diagnoses when necessary. The non-patient version of SCID-I (47) sections for mood, psychotic, and substance abuse, as well as the SCID II, was used to identify controls without a history of psychiatric or personality disorders. The positive and negative syndrome scale (PANSS) (48) was used to rate clinical symptoms in the schizophrenia group. A total of 53 of the 56 participants in the schizophrenia group had PANSS scores available within 2 weeks of the time of dEBC testing.

Participants were excluded from the experiment if they had clinically significant hearing loss, cardiovascular disease, an intelligence quotient (IQ) score of less than 70, had received electroconvulsive therapy, or if they had a history of neurological disorders, head injury resulting in loss of consciousness, or alcohol or substance dependence within the 3 months prior to their participation in the experiment. Additional exclusion criteria for potential control group participants were history of psychotic or mood disorders, or having a first-degree relative with a schizophrenia spectrum diagnosis. All aspects of this study were approved by the Indiana University Human Subjects Institutional Review Board (IUB-IRB; Protocol #1009001702), and all participants provided written informed consent prior to participation in the study.

### Delay Eyeblink Conditioning Procedure

The experiment consisted of 10 blocks of dEBC, with 10 trials per block. Of these 10 trials, 9 were paired with a conditioned stimulus tone lasting 400 ms (1000 Hz, 80 dB) that co-terminated with a 50 ms unconditioned stimulus air puff (10 psi at the source). A single tone-alone trial was also randomly presented during each block. The experiment began with eight unconditioned stimuli (15 s average inter-trial interval with a range of 10–20 s) that were presented alone to assess the integrity of eyeblink responses. Participants rated neutral pictures from the International Affective Picture System (49) throughout the experiment to maintain alertness. Pictures were presented for 2 s between trials and participants indicated the pleasantness of each picture on a response pad. Participants were monitored using a closed circuit camera to ensure their eyes remained open during the experiment. In cases in which a participant’s eyes appeared to close, the experiment was briefly suspended so alertness could be re-established by turning on the lights and offering the participant a drink of water.

### Procedure

Electromyographic activity was recorded from the orbicularis palpebrarum of the left eye by placing two bipolar electrodes 1 cm below the left eyelid, approximately 1 cm apart, and centered beneath the pupil. A ground electrode was placed on the forehead. The 50 ms unconditioned stimulus air puff was delivered to the left eye via copper tubing affixed to lens-less glasses and connected to plastic tubing (approximately 120″) connected to a regulator. Ear inserts (E-A-RLINK – Aearo Company Auditory Systems) were used to deliver the conditioned stimulus tone. Electromyographic recordings were continuously recorded (2.5 kHz A/D rate; high-pass filter = 1 Hz; low-pass filter = 500 Hz; gain = 1000) and stored offline for further processing.

### Data Processing

The continuous dEBC data files were segmented into 1086 ms epochs starting 500 ms before the conditioned stimulus onset. Data were high-pass filtered using a 28 Hz (6 dB per octave) filter, rectified, then smoothed using a 41 point Gaussian weighted moving average. The 90 paired dEBC trials from each experiment were analyzed using DataMunch, a MatLab program specifically designed for eyeblink conditioning data analysis (36, 38–40, 44, 45, 50–52). Blinks that occurred between 25 and 100 ms were characterized as alpha responses, which occur in response to the conditioned response tone onset and are reflexive, orienting responses that are not learning-related phenomena. For each participant, eyeblinks were counted as conditioned responses if they exceeded 5 SDs of baseline activity (baseline = 125 ms prior to conditioned stimulus onset) for each trial.

Trials in which electromyographic activity increased during the time window beginning 25 ms prior to the conditioned stimulus onset through 75 ms post-onset were excluded from analysis. These trials were excluded because blinks during this interval are not considered learning-related, and can interfere with the emission of a true conditioned response eyeblink.

Conditioned responses were recorded when an eyeblink occurred between 100 and 350 ms after the tone’s onset, the time interval corresponding to the 250 ms prior to the unconditioned stimulus onset. The onset latency was calculated as the time when the electromyographic activity exceeded 0.5 SDs from baseline activity.

### Statistical Analysis

Block-by-block percentages of conditioned responses from dEBC experiments were fitted to growth curve models using HLM. Conditioned response averages for each of the 10 blocks for each individual were calculated and the best-fitting line was generated, resulting in one line for each participant – a total of 154 lines. Eleven from this initial group (six participants with schizophrenia and five controls) were dropped from the analysis because they failed to exhibit conditioned responding such that the difference between the last and the first estimation of a linear curve fit was <0%. Therefore, 143 participants remained for age-matching (60 in the schizophrenia group; 83 in the control group). The final sample included 59 participants with schizophrenia who were age-matched to a healthy control whose age was within 2 years of their own.

The lme function of the nlme package (53) in R 3.0 (R Development Core Team, 2009) was used to model associative learning for growth curve modeling in HLM. Models used maximum likelihood estimation, except when testing whether effects should be fixed or random, in which case restricted maximum likelihood was used as suggested by Singer and Willett (54). Linear and non-linear forms of change were examined with nested model comparisons using the likelihood ratio test. Model fit was examined with pseudo-*R*^2^ (54), which was calculated by the squared correlation between the model’s fitted and observed values, representing the proportion of variance in the outcome explained by the model.

A three-parameter logistic growth curve with a randomly varying asymptote and fixed values for the slope and inflection point was used, which fit the data well (pseudo-*R*^2^ = 0.73). The model allowed different asymptote estimates across participants but not different estimates of slope or inflection point (but were allowed to differ by group). A random effect of asymptote was a better model fit than a model with a random effect of inflection point, and models with a random effect of slope did not converge. For each individual, logistic growth curves were fit to associative learning curves across the 10 blocks of the experiment. These logistic curves estimated whether the groups were different for each of the three parameters: slope, inflection point, and asymptote. The inflection point is the point on the curve where it changes curvature, and the asymptote is where learning begins to level off. The slope measures the change in associative learning over time and was used to assess differences in learning rate between groups.

We attempted to analyze data from conditioned response onset latency, but the data fit a logistic growth curve model poorly (pseudo-*R*^2^ = 0.24). Therefore, although all indications were that no differences on primary dependent variables could be observed, given the lack of fit and consequent unreliability of statistical measures, we have not included this analysis in the Section “Results.”

Using three separate statistical tests of between-group differences (schizophrenia vs. controls for asymptote, slope, and inflection point), a Bonferroni-corrected alpha level of *P* < 0.017 (*P* < 0.05/3 comparisons) was deemed significant, although results with *P* < 0.05 are reported.

## Results

### Baseline Unconditioned Response Amplitude

Differences in conditioned response measurements could arise from impairment in general eyeblink performance. Therefore, to ensure that any observed differences between groups on the percentage of conditioned responses was not due to such a general performance issue, eight unconditioned stimulus air puffs were presented alone at the beginning of the experiment. Baseline unconditioned response amplitude was available for a total of 41 participants with schizophrenia and 42 controls. Neither the average peak unconditioned response amplitudes [*F*(1,81) = 3.17, *P* = 0.08] nor latencies [*F*(1, 81) = 0.003, *P* = 0.96] were significantly different between groups. While the differences in amplitude did not reach significance, it is important to note that average group differences indicated that the schizophrenia group had *larger* unconditioned response amplitudes (*M* = 97.89 μV, SD = 23.27) compared to controls (*M* = 89.64 μV, SD = 18.79). This finding is consistent with earlier findings that unconditioned response amplitude was larger on paired dEBC trials in schizophrenia (40). Overall, these findings suggest that differences in conditioned responses are unlikely to be due to deficits in blink performance in the schizophrenia group.

### Percentage of Conditioned Responses

**Figure 1:**
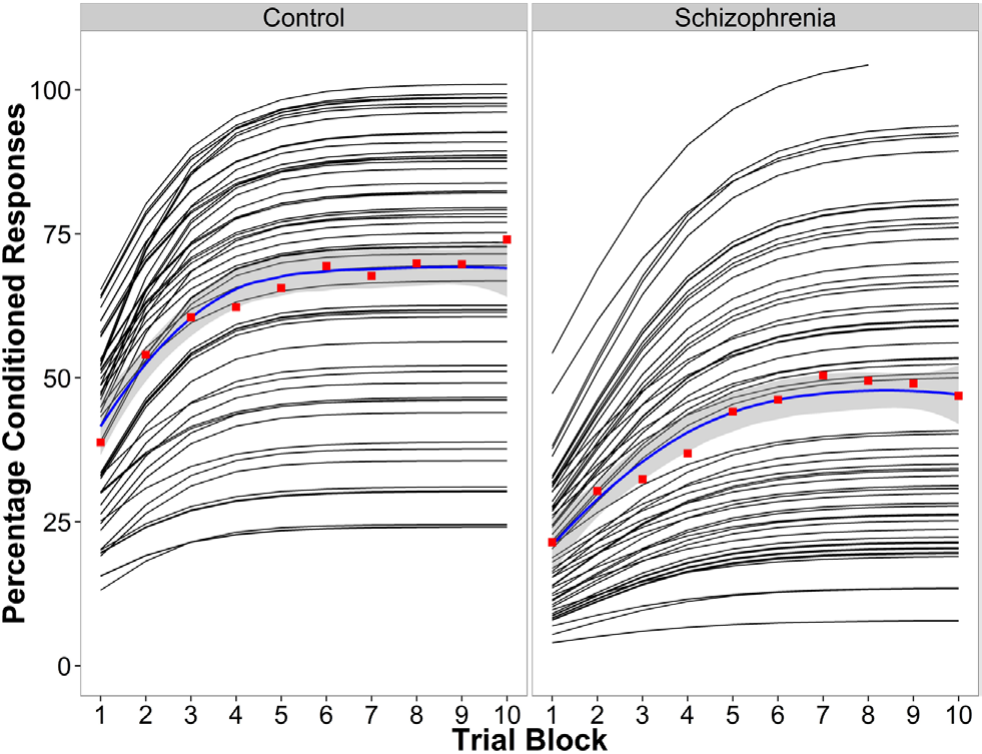
**Conditioned response data for the control group (left) and the schizophrenia group (right)**. The logistic curve fit for each individual (black lines), the average percentage of CRs for the raw data for each block in red, and the group average logistic curve fit in blue.

Parameter estimates of the logistic model examining learning curves of the percentage of conditioned responses are in Table 2. Figure 1 shows the line fits for each participant, the group average fitted line, and the conditioned response average for each of the 10 blocks. Findings suggest that the difference in learning between the beginning and end of the experiment is similar between groups, but that learning saturates later in the schizophrenia group, and the level at which saturation occurs is lower in the schizophrenia group. When the groups were considered together, performance improved across the 10 blocks of the experiment, *t*(1003) = 5.88, *P* < 0.001, SE = 0.19, and the rate of learning did not differ between groups, *t*(1003) = 1.59, *P* = 0.11, SE = 0.33. However, the asymptote was significantly lower in the schizophrenia group, *t*(1003) = −4.09, *P* < 0.001, SE = 4.97. Moreover, the inflection point occurred later in schizophrenia group [*t*(1003) = 2.77, *P* = 0.006, SE = 0.27]. These results indicate that the rate of learning over the course of the experiment (the slope), measured as the difference between blocks 1 and 10 on the fitted logistic curves, was not significantly different between groups. However, the reduced asymptote in schizophrenia makes the slope more similar between groups even though the inflection point occurred later. Overall, the schizophrenia group attained a lower ultimate level of learning and took longer to achieve this maximum.

**Table 2:** **Parameter estimates for the HLM growth curve model for percentage of conditioned responses**.

	Value (SE)	DF	*t*-value	*p*-value
*R*^2^ = 0.73				
Asymptote	68.92 (3.46)	1003	−19.91	0.000
SZ-HC	−20.31 (4.97)	1003	−4.09	0.000*
Inflection Point	0.64 (0.17)	1003	3.59	0.000
SZ-HC	0.74 (0.27)	1003	2.77	0.006*
Slope	1.1 (0.19)	1003	5.88	0.000
SZ-HC	0.51 (0.33)	1003	1.59	0.112

*SZ, schizophrenia, HC, healthy controls*.

*Indicates differences between groups with a significance at *P* < 0.017.

### Correlations with Clinical Symptoms

We examined associations of participants’ estimates on each of the three logistic model parameters for the percentage of conditioned responses with PANSS positive, negative, general, and total scores using bivariate correlations with age partialed out. There were no significant correlations between any behavioral parameters and clinical variables.

## Discussion

The goal of the present study was to extend and clarify results of earlier studies examining dEBC in schizophrenia using more sophisticated statistical models. HLM of data fitted to a logistic growth curve model provided insight into how three components of the learning curve change over time in schizophrenia. Overall, associative learning in the schizophrenia group leveled off at a lower level compared to controls, and took longer to reach the maximal learning level. Surprisingly, the rate of learning (i.e., slope) within subjects with schizophrenia was not significantly different from controls.

Analysis of dEBC data using HLM, a superior analytic approach compared to ANOVA, suggests robust differences between subjects in the control and schizophrenia groups. Cerebellar abnormalities in schizophrenia are most likely responsible for these behavioral dEBC differences. The regions of cerebellar cortex that show reduced regional cerebral blood flow (rCBF) during dEBC in unmedicated schizophrenia (55) also overlap with those identified as fundamental to normal expression of conditioned eyeblink responses in animal studies (56–59). The interpositus nucleus is necessary for the acquisition and retention of the conditioned eyeblink response with cerebellar cortical sites, in particular long-term depression at the parallel fiber–Purkinje cell synapse, modulating important aspects of the gain and timing of the response (see, Ref. (60) for extensive review). Human studies of populations with cerebellar lesions or degeneration largely support these findings, and also suggest that purely cortical lesions produce significant reductions in the expression of conditioned responses, but do not abolish them (61). Importantly, cerebellar cortical structure is associated with conditioned response timing (62) and acquisition (63). Taken together, these findings suggest that abnormalities in the interpositus nuclei and the cortex of the cerebellum contribute to the dEBC deficits observed in schizophrenia.

Our laboratory has undertaken a program of research that aims to tackle outstanding questions about cerebellar abnormalities in schizophrenia. We have previously reported deficits in schizophrenia on timing tasks that rely heavily on cerebellar-based timing mechanisms, including paced finger-tapping (64) and a temporal bisection task (45, 65, 66). Using neuroimaging techniques, we can more definitively understand the extent to which the dEBC deficits in schizophrenia are uniquely attributable to alterations in cerebellar function compared to other cortical and subcortical circuits in which the cerebellum participates. We are currently using functional magnetic resonance imaging in conjunction with dEBC and paced finger-tapping to determine how cerebellar functional and structural abnormalities contribute to performance deficits in schizophrenia. Moreover, our recent studies have identified dEBC abnormalities in an intermediate phenotype of schizophrenia, namely schizotypal personality disorder (39), and in first-degree relatives of individuals with schizophrenia (44), suggesting that dEBC impairments may be risk markers for schizophrenia. Ongoing studies of first-degree relatives will determine whether familial risk is associated with morphological and functional alterations in the cerebellum and related circuits.

Our current studies and others addressing similar questions may provide evidence that the cerebellum is a potential therapeutic target for remediating symptoms of schizophrenia. Indeed, preliminary evidence supports this idea. For example, secretin is a neuropeptide with receptors in the cerebellum, which permitted us to make predictions based on a mechanistic model of its actions within the cerebellar cortex (67, 68). When we administered secretin to a small group of participants with schizophrenia, it significantly improved dEBC performance and validated the utility of the cerebellum as a potential pharmacological target (37) [c.f., Ref. (69, 70)]. Similarly, a small sample of individuals with treatment-resistant schizophrenia underwent theta-burst transcranial magnetic stimulation of the cerebellum and experienced both improved mood symptoms and enhanced cognitive performance (71). Taken together, efforts to identify cerebellar-dependent biomarkers will facilitate the development of new potential therapeutic targets within the cerebellum that could provide previously unexplored avenues of treatment that are sorely needed for this perplexing disorder.

## Conflict of Interest Statement

The authors declare that the research was conducted in the absence of any commercial or financial relationships that could be construed as a potential conflict of interest.
